# Supplementary material for: Ferromagnetism above 1000 K in a highly cation-ordered double-perovskite insulator Sr3OsO6
Source: Nat Commun. 2019 Feb 12;10:535. doi: 10.1038/s41467-019-08440-6 (PMC6372617; doi:10.1038/s41467-019-08440-6)
Supplement: Supplementary file 1 — Supplementary Information [file 41467_2019_8440_MOESM1_ESM.pdf]

Supplementary Information

Ferromagnetism above 1000 K in a highly cation-ordered double-perovskite insulator Sr_3OsO_6

Wakabayashi et al.

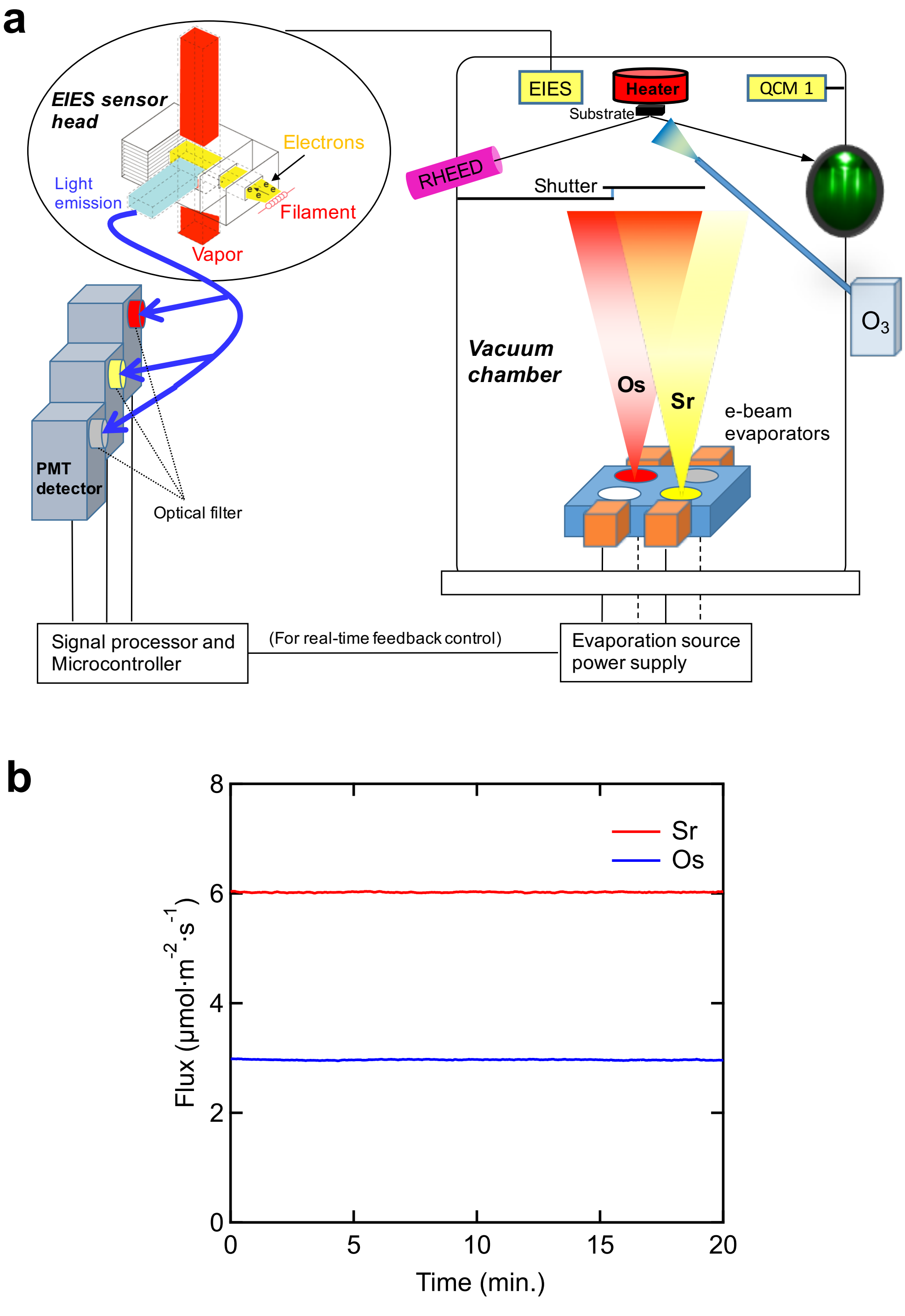

Supplementary Figure 1 Multi-source oxide MBE setup and fluxes. a, Schematic illustration of our multi-source oxide MBE setup. EIES: electron impact emission spectroscopy. QCM: quartz crystal microbalance. RHEED: reflection high-energy electron diffraction. PMT: photomultiplier tube. **b**, Sr and Os fluxes measured by EIES during the growth.

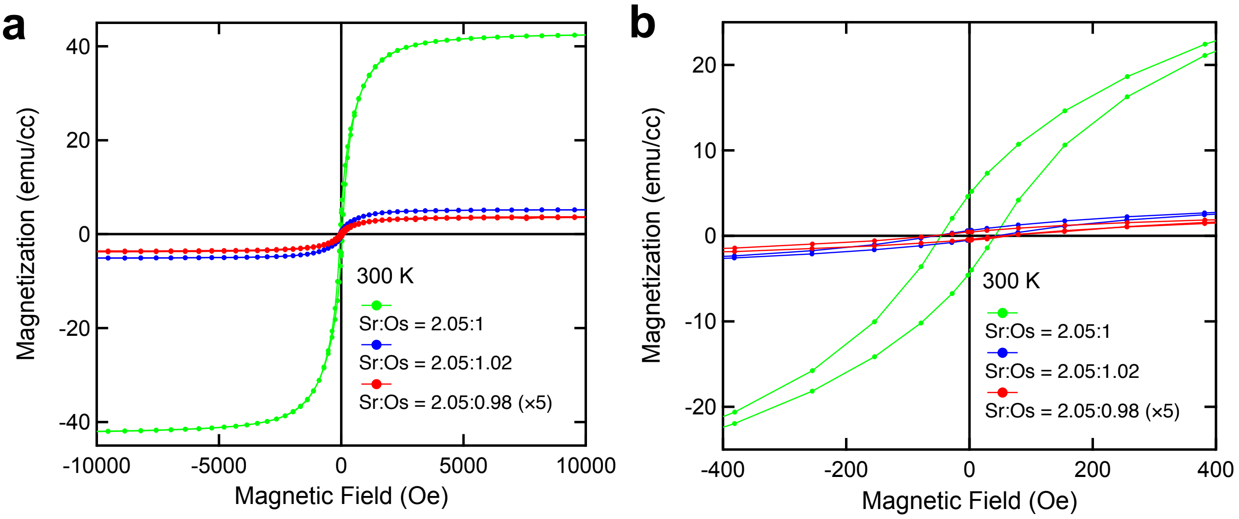

Supplementary Figure 2 Magnetic properties of Sr_3OsO_6 films grown with different flux ratios of Sr and Os. a, In-plane *M*-*H* curves at 300 K for Sr_3OsO_6 films grown with different flux ratios of Sr and Os. Here, *H* was applied to the [100] direction. **b**, Close-up near the zero magnetic field in **a**.

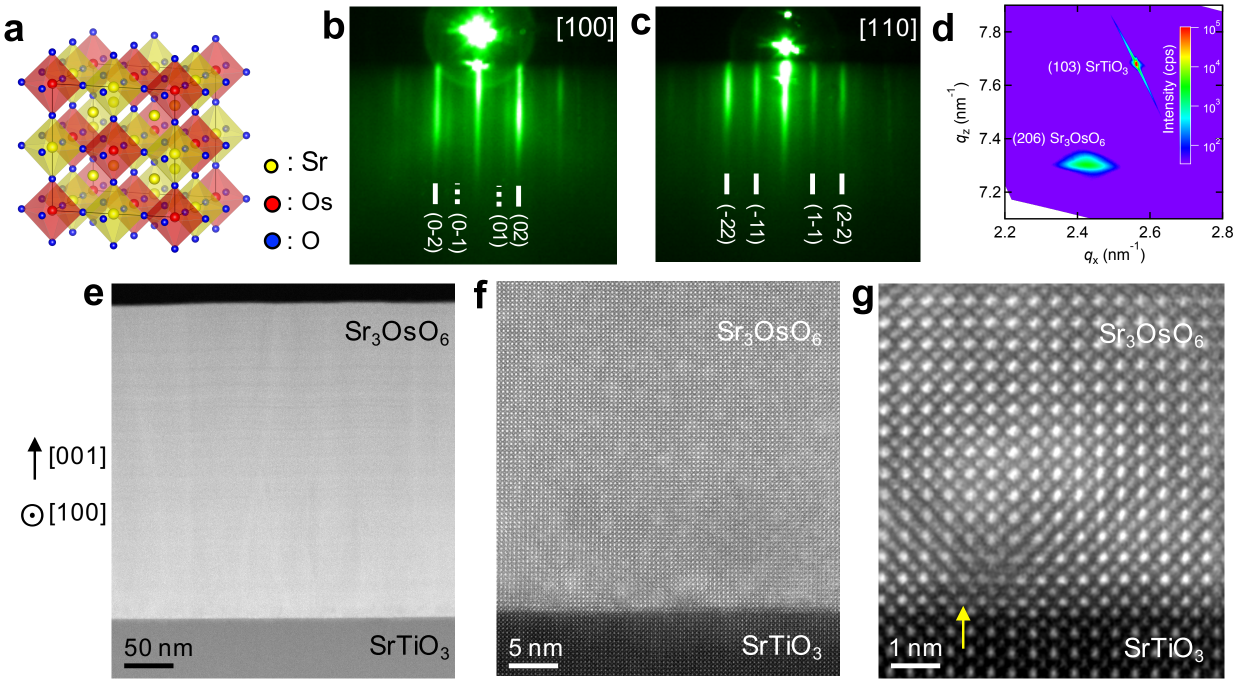

Supplementary Figure 3 Crystal structure, RHEED, X-ray HRRSM and HAADF-STEM for a Sr_3OsO_6 film. a, Schematic diagram of the B-site ordered double-perovskite Sr_3OsO_6. The yellow, red and blue spheres indicate Sr, Os and O ions, respectively. **b**, **c,** RHEED patterns of an epitaxial Sr_3OsO_6 film surface, where the incident electron beams are parallel to [100] (**b**) and [110] (**c**). **d**, X-ray HRRSM of a Sr_3OsO_6 film around the $SrTiO_3$ (103) reflection. **e,** Cross-sectional HAADF-STEM images of a Sr_3OsO_6 film taken along the [100] direction. **f,** Magnified image near the interface in **e**. **g,** Magnified image near the interface in **f**. In **g**, a misfit dislocation at the Sr_3OsO_6/$SrTiO_3$ interface is indicated by the yellow arrow.

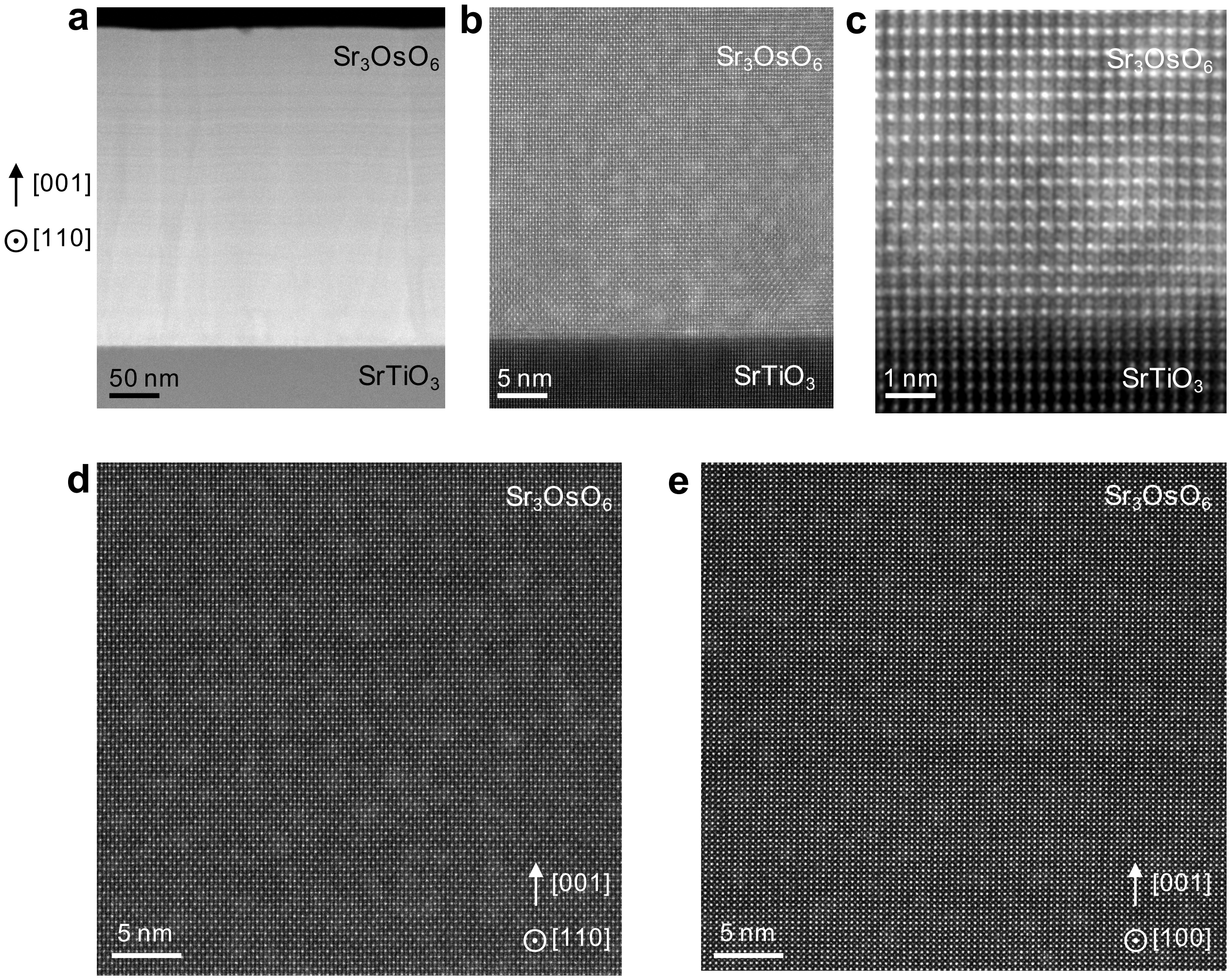

Supplementary Figure 4 STEM images for a Sr_3OsO_6 film. a, Cross-sectional HAADF-STEM image of a Sr_3OsO_6 film taken along the [110] direction. **b,** Magnified image near the interface in **a**. **c,** Magnified image near the interface in **b**. **d, e**, HAADF-STEM images of the Sr_3OsO_6 layer taken along the [110] (**d**) and [100] (**e**) directions.

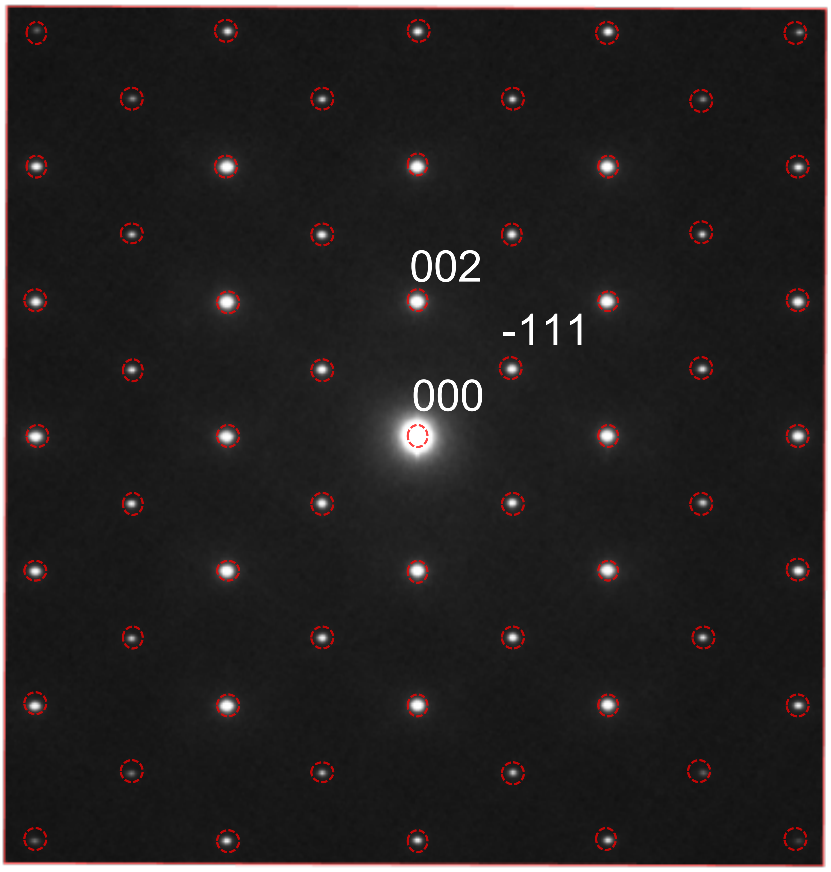

Supplementary Figure 5 TED pattern for a Sr_3OsO_6 film. TED pattern for a Sr_3OsO_6 film taken along the [110] axis. The red dashed circles represent calculated diffraction pattern obtained by the Fourier transform of the ideal cubic B-site ordered double-perovskite structure.

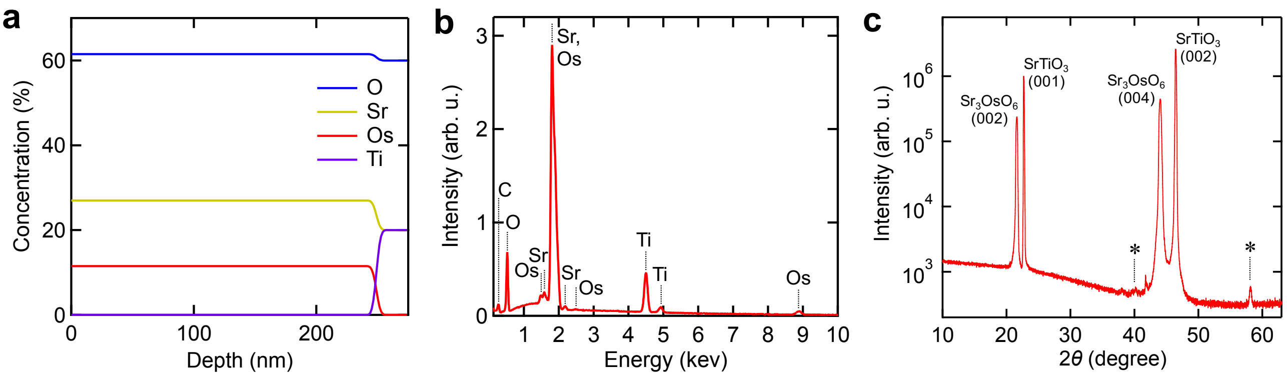

Supplementary Figure 6 Chemical composition, EDS spectrum and θ-2θ XRD pattern of Sr_3OsO_6 films. **a**, Depth profile of the chemical composition of a Sr_3OsO_6 film (250-nm thick) estimated from RBS. **b**, EDS spectrum of a Sr_3OsO_6 film, which was taken from a wide area (1×1 mm^2). **c**, θ-2θ XRD pattern for a Sr_3OsO_6 film. Traces of OsO_2 are detected as indicated by $*$.

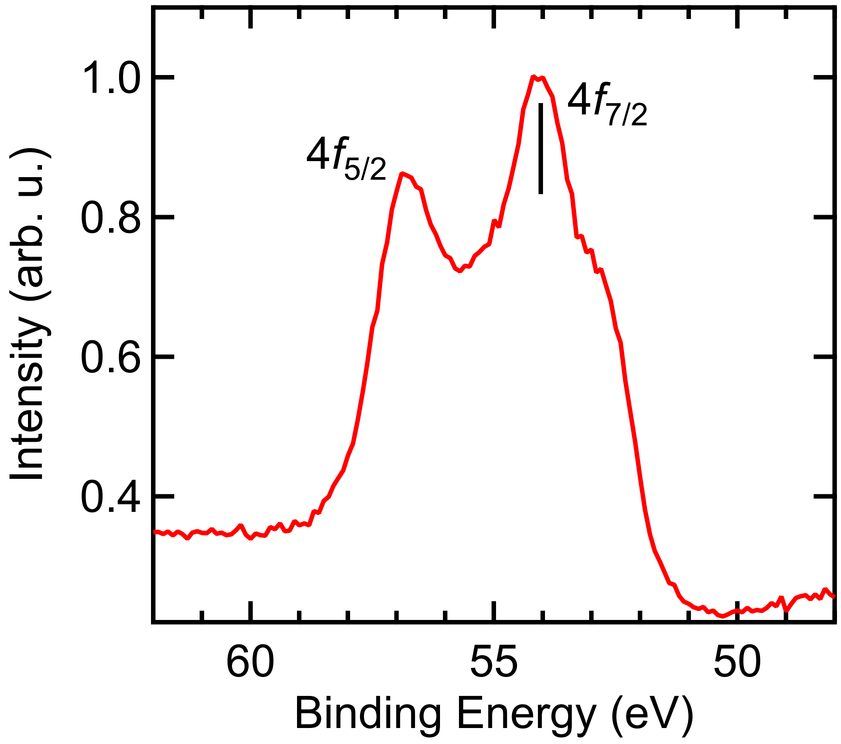

Supplementary Figure 7 XPS of a Sr_3OsO_6 film. The Os 4*f* XPS spectrum of a Sr_3OsO_6 film at 300 K.

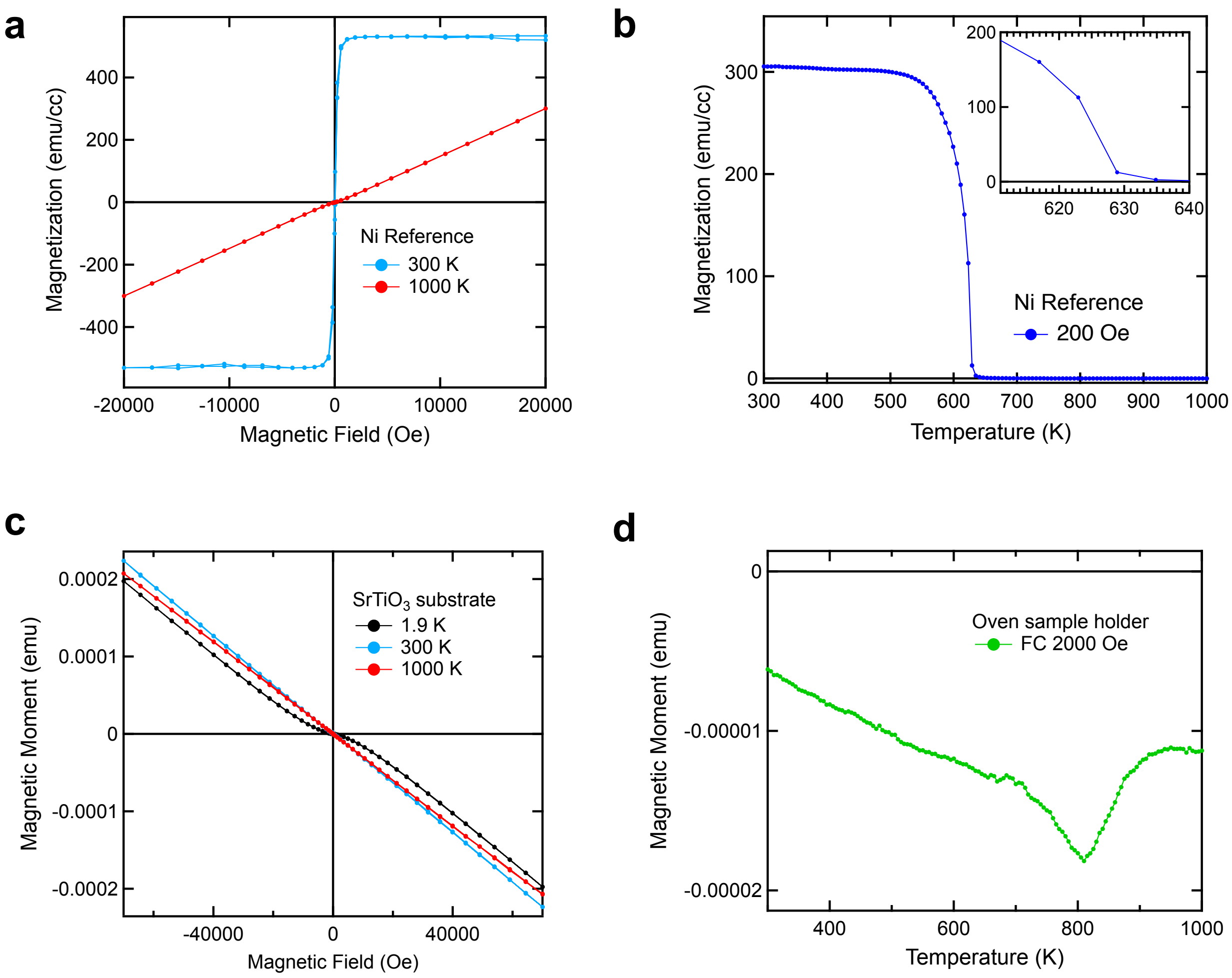

Supplementary Figure 8 Magnetic properties of a Ni reference plate and a $SrTiO_3$ substrate, and experimental artifact from oven sample holder. a, *M-H* curve at 300 and 1000 K for a Ni plate. Here, *H* was applied to the in-plane direction. **b**, *M-T* curve with *H* = 200 Oe applied to the in-plane direction for a Ni plate. The inset of **b** shows a close-up near the Curie temperature. **c**, In-plane *M-H* curve at 1.9, 300 and 1000 K for a $SrTiO_3$ substrate. Here, *H* was applied to the [100] direction. **d**, In-plane *M-H* curve at *M-T* curve with *H* = 2000 Oe for the oven sample holder without a sample.

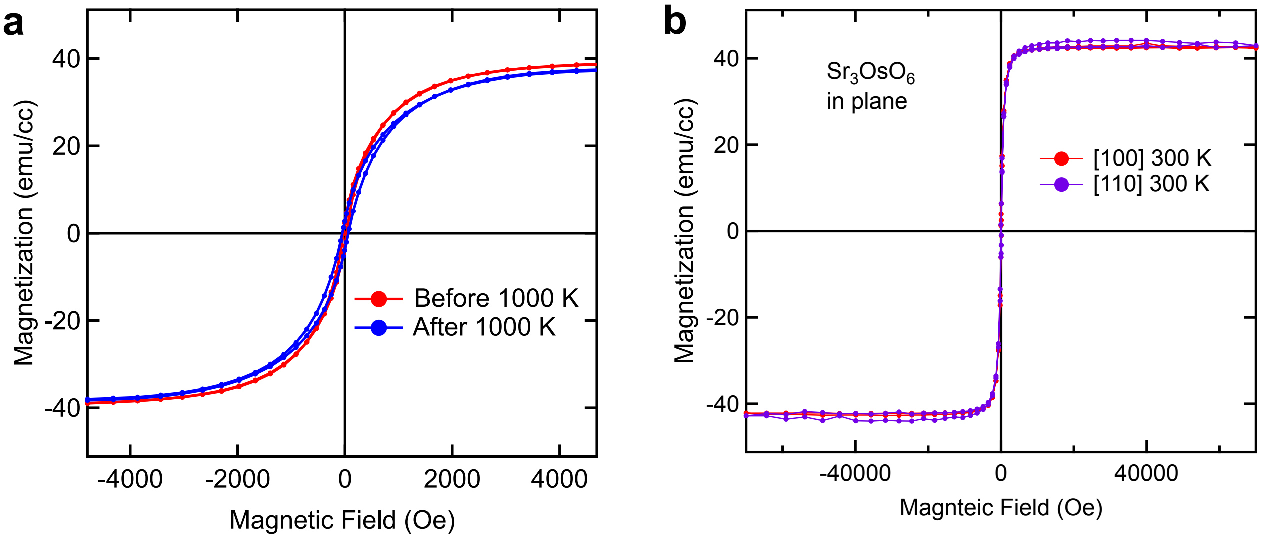

Supplementary Figure 9 Magnetic properties of a Sr_3OsO_6 film after heating and magnetic properties of a Sr_3OsO_6 film with *H* applied to the [100] and [110] directions. a, In-plane *M*-*H* curves of a Sr_3OsO_6 film at 300 K before and after the sample was heated to 1000 K. Here, *H* was applied to the [100] direction. **b**, In-plane *M*-*H* curves at 300 K for a Sr_3OsO_6 film. Here, *H* was applied to the [100] or [110] direction.

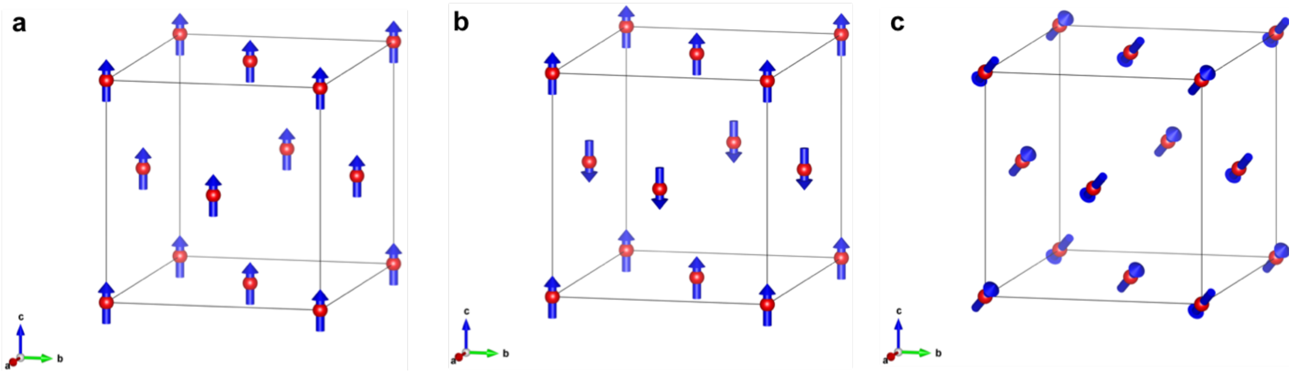

Supplementary Figure 10 Schematic diagrams of the magnetic orders. a, **b**, **c,** Schematic diagram of the collinear FM order (**a**), the (001) AFM order (**b**) and the (111) AFM order (**c**). In the diagrams, red spheres and blue arrows indicate Os atoms and Os magnetic moments, respectively, and the Sr and O atoms are omitted for simplicity.

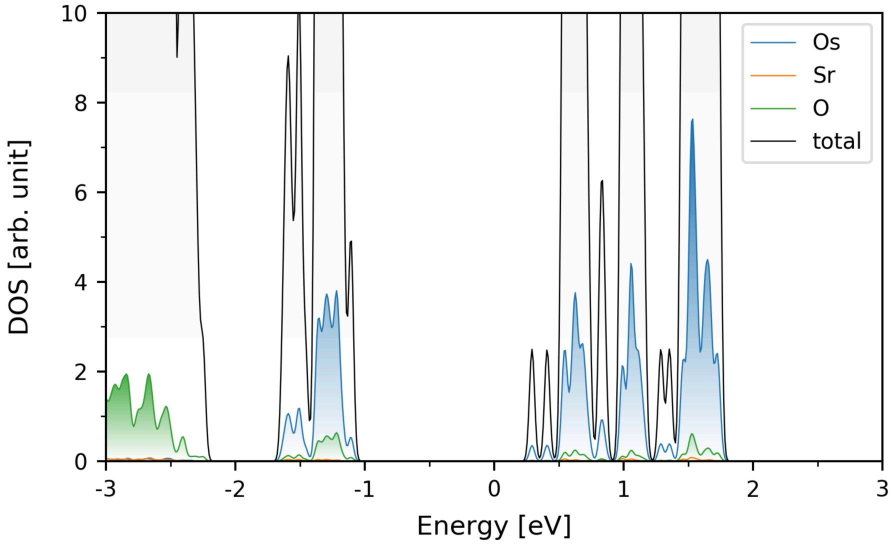

Supplementary Figure 11 Electronic structure calculation with the HSE functional. The element-specific PDOS for the canted FM order calculated by the HSE + SOC method. Here, blue, orange and green curves indicate the density-of-states per one Os, Sr and O atoms, respectively.
